# Insecticide resistance status of three malaria vectors, *Anopheles gambiae* (*s.l.*), *An*. *funestus* and *An*. *mascarensis*, from the south, central and east coasts of Madagascar

**DOI:** 10.1186/s13071-017-2336-9

**Published:** 2017-08-23

**Authors:** Jean-Desire Rakotoson, Christen M. Fornadel, Allison Belemvire, Laura C. Norris, Kristen George, Angela Caranci, Bradford Lucas, Dereje Dengela

**Affiliations:** 1 President’s Malaria Initiative Africa Indoor Residual Spraying Project, Abt Associates, Antananarivo, Madagascar; 2 U.S. President’s Malaria Initiative, United States Agency for International Development, Bureau for Global Health, Office of Infectious Disease, 2100 Crystal Drive, Arlington, VA 22202 USA; 3 President’s Malaria Initiative Africa Indoor Residual Spraying Project, Abt Associates, 4550 Montgomery Ave, Suite 800 North, Bethesda, MD 20814 USA

**Keywords:** Madagascar, Insecticide resistance, *An*. *gambiae* (*s.l.*), *An*. *funestus*, *An*. *mascarensis*

## Abstract

**Background:**

Insecticide-based vector control, which comprises use of insecticide-treated bed nets (ITNs) and indoor residual spraying (IRS), is the key method to malaria control in Madagascar. However, its effectiveness is threatened as vectors become resistant to insecticides. This study investigated the resistance status of malaria vectors in Madagascar to various insecticides recommended for use in ITNs and/or IRS.

**Methods:**

WHO tube and CDC bottle bioassays were performed on populations of *Anopheles gambiae* (*s.l.*), *An*. *funestus* and *An*. *mascarensis.* Adult female *An*. *gambiae* (*s.l.*) mosquitoes reared from field-collected larvae and pupae were tested for their resistance to DDT, permethrin, deltamethrin, alpha-cypermethrin, lambda-cyhalothrin, bendiocarb and pirimiphos-methyl. Resting *An*. *funestus* and *An*. *mascarensis* female mosquitoes collected from unsprayed surfaces were tested against permethrin, deltamethrin and pirimiphos-methyl. The effect on insecticide resistance of pre-exposure to the synergists piperonyl-butoxide (PBO) and S,S,S-tributyl phosphorotrithioate (DEF) also was assessed. Molecular analyses were done to identify species and determine the presence of knock-down resistance (*kdr*) and acetylcholinesterase resistance (*ace-1* ^*R*^ *)* gene mutations.

**Results:**

*Anopheles funestus* and *An*. *mascarensis* were fully susceptible to permethrin, deltamethrin and pirimiphos-methyl. *Anopheles gambiae* (*s.l.*) was fully susceptible to bendiocarb and pirimiphos-methyl. Among the 17 *An*. *gambiae* (*s.l.*) populations tested for deltamethrin, no confirmed resistance was recorded, but suspected resistance was observed in two sites. *Anopheles gambiae* (*s.l.*) was resistant to permethrin in four out of 18 sites (mortality 68–89%) and to alpha-cypermethrin (89% mortality) and lambda-cyhalothrin (80% and 85%) in one of 17 sites, using one or both assay methods. Pre-exposure to PBO restored full susceptibility to all pyrethroids tested except in one site where only partial restoration to permethrin was observed. DEF fully suppressed resistance to deltamethrin and alpha-cypermethrin, while it partially restored susceptibility to permethrin in two of the three sites. Molecular analysis data suggest absence of *kdr* and *ace-1* ^*R*^ gene mutations.

**Conclusion:**

This study suggests involvement of detoxifying enzymes in the phenotypic resistance of *An*. *gambiae* (*s.l.*) to pyrethroids. The absence of resistance in *An*. *funestus* and *An*. *mascarensis* to pirimiphos-methyl and pyrethroids and in *An*. *gambiae* (*s.l.*) to carbamates and organophosphates presents greater opportunity for managing resistance in Madagascar.

**Electronic supplementary material:**

The online version of this article (doi:10.1186/s13071-017-2336-9) contains supplementary material, which is available to authorized users.

## Background

Intensive vector control efforts have led to a dramatic decline in the global malaria burden in the past decade [1]. According to the World Health Organization’s (WHO) World Malaria Report 2015, malaria morbidity has decreased from an estimated 262 million in 2000 to 214 million in 2015, a reduction of 18% over the 15 years [2]. Four major malaria intervention methods are credited for the significant malaria control gains: long-lasting insecticidal nets, indoor residual spraying (IRS), diagnosis and management of malaria cases, and intermittent preventive treatment in pregnancy [2]. However, the emergence and spread of resistance in malaria vectors and parasite populations to insecticides and artemisinin, respectively, is threatening to slow and even reverse the gains made in malaria control. Insecticide resistance in the major malaria vectors, *Anopheles gambiae* (*s.s.*), *An*. *arabiensis*, *An*. *coluzzii* and *An*. *funestus*, has been reported from east [3–8], west [3, 9–16], central [3, 17–20] and southern Africa [3, 21–23]. Artemisinin resistance in *Plasmodium falciparum* has been reported from five countries, all in southeast Asia (Cambodia, the Lao People’s Democratic Republic, Myanmar, Thailand and Vietnam) [2].

If hard-won malaria control gains are to be sustained and further progress made toward the long-term goal of a malaria-free world, malaria control communities need to preserve the efficacy of existing tools even as they pursue innovative methodologies. In 2012, WHO developed and launched the Global Plan for Insecticide Resistance Management in coordination with the Roll Back Malaria partnership, to help guide and improve the planning and implementation of resistance management strategies in malaria-endemic countries [24].

Nearly all of Madagascar’s population is at risk of contracting malaria [25]. *Anopheles gambiae* (*s.s.*) is the primary vector on the east and west coasts, where malaria prevalence is stable and transmission lasts longer than six months per year [26]. *Anopheles funestus* and *Anopheles arabiensis* are secondary vectors in these coastal areas [25]. In the south sub-desert and central highlands (CHL), where *An*. *funestus* is the primary vector and *An*. *arabiensis* and *An*. *mascarensis* are secondary vectors [27], malaria is seasonal and prone to epidemics. *Anopheles mascarensis* is implicated as the primary and secondary vector in the South-East sub-desert and Sainte Marie District, respectively [28]. A recent study also implicated *An*. *coustani* involvement in the transmission of malaria in the CHL [29].

Malaria vector control with IRS has a long history in Madagascar. IRS with DDT started in 1949 [29]. Impressive results were achieved in the 1970s when this intervention was coupled with malaria case management using chloroquine [30]; malaria transmission was interrupted and *An*. *funestus* was eliminated from most parts of the CHL, and malaria morbidity significantly declined even in the perennial transmission areas. After IRS was stopped in 1979, *An*. *funestus* gradually re-emerged in the CHL as did an increased occurrence of malaria epidemics in 1986 [30]. In response to the resurgence of malaria transmission in the CHL, in 1993, IRS with DDT was re-introduced [30]. Currently, the two major malaria vector control methods used in Madagascar are IRS and insecticide-treated nets (ITNs) [31]. Madagascar adopted the policy of universal coverage of ITNs in 2008. A malaria indicator survey conducted in 2013 showed that 79% of the target population had access to at least one ITN [31]. A malaria case-control survey conducted in 2013 estimated that IRS and ITNs each provided a protective efficacy of 51% when implemented individually [32]. When the two interventions were implemented in combination, a protective efficacy of 72% was recorded [32]. Any change in the response of malaria vectors to the insecticides used in the two interventions could negatively affect malaria control efforts.

*Anopheles gambiae* (*s.l.*) resistance to DDT was first observed in Madagascar in the late 1990s, and appeared to be widespread in the CHL in the early 2000s [33, 34]. Initial evidence of resistance to permethrin was reported, but local populations of *An*. *gambiae* (*s.l.*) remained fully susceptible to lambda-cyhalothrin, deltamethrin, cyfluthrin and alpha-cypermethrin [33, 34], while *An*. *funestus* was fully susceptible to DDT and pyrethroids [33, 34]. However, these studies, conducted between 1996 and 2003, were limited in geographical scope to the CHL, and no updated resistance data have been published since 2003, despite the scale-up of insecticide-based vector control in Madagascar in the past decade. More recent studies from other countries have reported rapid changes in resistance patterns following increased use of insecticides for public health and/or agriculture [10–13]. For all these reasons, regular resistance monitoring from nationally representative sites is warranted to inform the planning and implementation of successful vector control and resistance management strategies.

The objective of the current study was to assess and report on the insecticide resistance profiles of three malaria vectors known to transmit the disease in Madagascar: *An*. *gambiae* (*s.l.*), *An*. *funestus* and *An*. *mascarensis*. The assessment was based on phenotypic resistance and resistance mechanisms. There has been no data published on the efficacy of public health insecticides since 2003; the previous studies covered only one out of five eco-epidemiological zones (the central highlands) and two classes of insecticides (pyrethroids and DDT). The present study will help to fill some of the information gap and for the first time report on the susceptibility of *An*. *mascarensis* to pyrethroids and pirimiphos-methyl.

## Methods

### Study area and duration

**Fig. 1:**
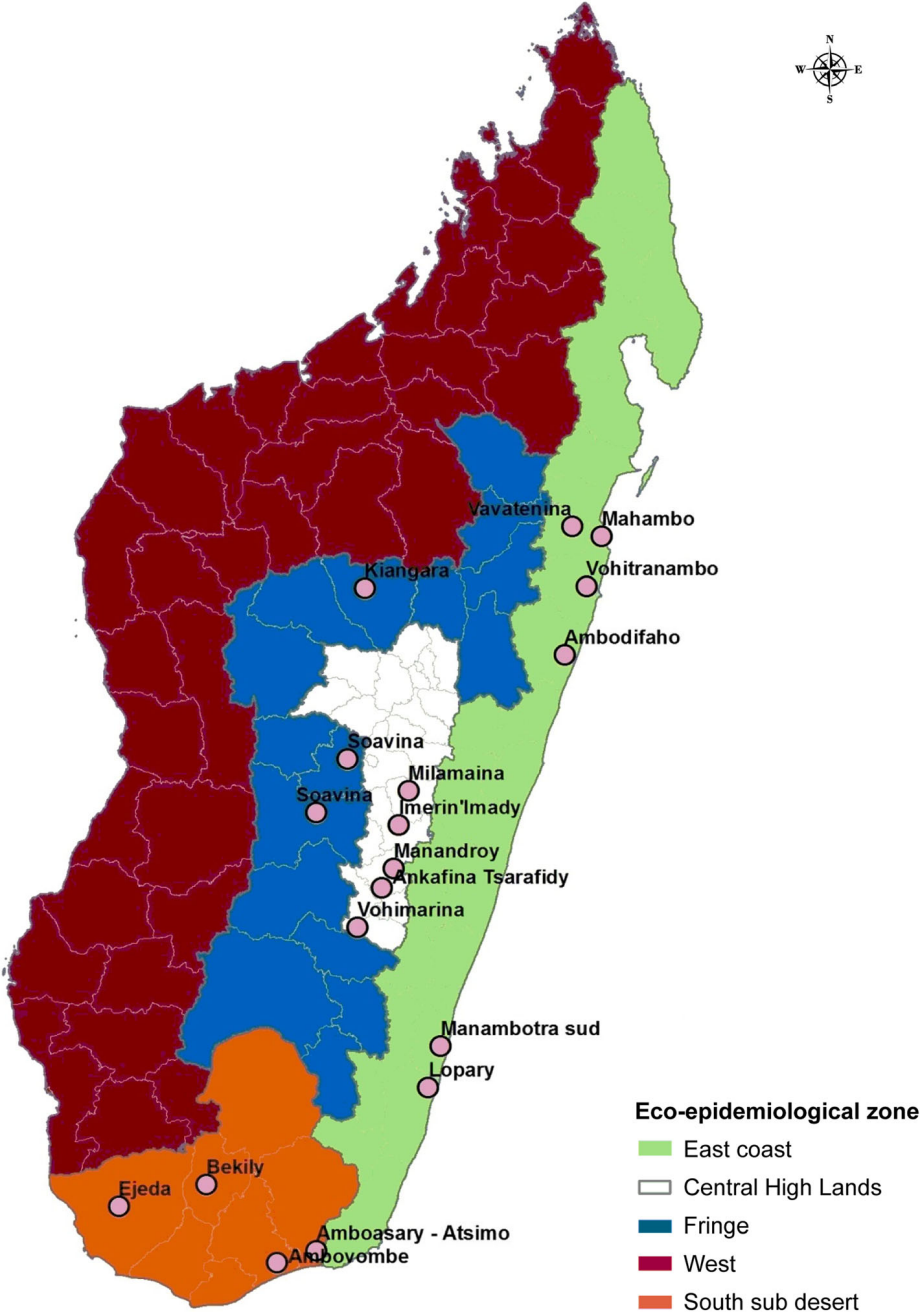
Insecticide resistance sites and malaria eco-epidemiological zones in Madagascar

In 2013/2014, the response of *An*. *gambiae* (*s.l.*) to various insecticides was assessed in nine localities belonging to three of the five eco-epidemiological zones of Madagascar: the CHL, the CHL fringe areas and the South East (Table 1, Fig. 1). Selection of resistance monitoring site/s was mainly linked to the history and status of IRS operations and the types of insecticide used for IRS, as well as the distribution of malaria vectors and representation of the different eco-epidemiological zones. In 2014/2015, IRS was expanded to the east coast, and so it was included in the insecticide resistance monitoring in 2015/2016 (Table 1, Fig. 1).

**ITable 1:** Insecticide resistance monitoring sites and their relevant characteristics

Epidemiological zone	Region	District	Commune/Village	Status and history of vector control	Year	
					2013/2014	2015/2016
Central highlands	Amoron'l Mania	Ambositra	Imerina Imady	1993–2004 DDT IRS, PY IRS 2005–2007, 2013 & 2014 no IRS since 2015	×	×
	Haute Matsiatra	Ambohimahasoa	Manandroy	1993–2004 DDT IRS, PY IRS 2005–2007, 2013 & 2014	×	–
	Amoron'l Mania	Fandriana	Milamaina	1993–2004 DDT IRS, PY IRS in 2005–2007, 2013 & 2014 and no IRS since 2015	–	×
	Haute Matsiatra	Ambohimahasoa	Ankafina Tsarafidy	1993–2004 DDT IRS, PY IRS in 2005–2007 & 2014 and no IRS since 2015	–	×
	Haute Matsiatra	Fianarantsoa II	Vohimarina	1993–2004 DDT IRS, PY IRS in 2005–2007 & 2014 and no IRS since 2015		×
Central highland fringe	Amoron’I Mania	Ambatofinandrahana	Soavina	1993–2004 DDT IRS, carbamate IRS from 2011 to 2013 and withdrawn in 2014, ITNs since 2010.	×	–
	Vakinankaratra	Betafo	Soavina	1993–2004 DDT IRS, PY IRS 2005–2007 & carbamate IRS 2013 & 2014	×	–
	Analamanga	Ankazobe	Kiangara	1993–2004 DDT IRS, PY IRS in 2005–2007 & carbamate IRS from 2011–2013. IRS withdrawn in 2014	×	–
South sub-desert	Androy	Ambovombe	Ambovombe	Carbamate IRS from 2011 to 2013, OP IRS in 2014 and no IRS after 2015, ITNs since 2007	×	–
	Atsimo Andrefana	Ampanihy	Ejeda	Carbamate IRS from 2011–2013, OP IRS in 2014 and no IRS after 2015, ITNs since 2007	×	–
	Anosy	Amboasary	Amboasary	Carbamate IRS from 2011–2013, OP IRS in 2014 and no IRS after 2015, ITNs since 2007	×	–
	Androy	Bekily	Bekily	Carbamate IRS from 2011–2013, OP IRS in 2014 and no IRS after 2015, ITNs since 2007	×	×
East coast	Atsinanana	Toamasina II	Vohitrambato	ITNs since 2007, OP IRS introduced in 2014	–	×
	Analanjirofo	Fenerive East	Mahambo	ITNs since 2007, OP IRS introduced in 2014	–	×
	Atsinanana	Brickaville	Ambodifaho	ITNs since 2007, OP IRS introduced in 2015	–	×
	Analanjirofo	Vavatenina	Vavatenina	ITNs since 2007, control area, No IRS	–	×
South-east	Atsimo Atsinanana	Farafangana	Manambotra Sud	ITNs since 2007, OP IRS introduced in 2015	–	×
	Atsimo Atsinanana	Vangaindrano	Lopary	ITNs since 2007, control area, No IRS	–	×

*Abbreviations*: IRS, Indoor residual spraying; PY, pyrethroid; OP, organophosphate; DDT, dichlorodiphenyltrichloroethane

### Mosquito sampling

#### Sampling *An. gambiae* (*s.l*.) 

Larvae and pupae were collected from several different types of natural breeding habitats in each of the insecticide resistance testing sites; collection used a standard dipper. The larvae were morphologically sorted into *Anopheles* spp. and *Culex* spp. *Anopheles* larvae and pupae were transported to temporary local field-rearing sites. The pupae were immediately transferred to beakers that contained water and placed in cages until the adults emerged. The larvae were pooled and provided with Tetramin baby fish food until pupation. Larval containers were checked daily and pupae were transferred to mosquito cages until their emergence into adults. Emerged adult mosquitoes were kept in a separate rearing room and fed with 10% sucrose solution. Non-blood-fed, two-to-three-day-old female *An*. *gambiae* (*s.l.*) mosquitoes were used for insecticide resistance tests.

#### Sampling* An. funestus* group and *An. mascarensis*

Due to the difficulty of obtaining a sufficient number of *An. funestus* and *An. mascarensis* from sampling of aquatic stages for the susceptibility tests, adult mosquitoes were collected from unsprayed living rooms between 06:00 and 10:00 h using battery-operated Prokopack aspirators [35] and flashlights. For the sprayed areas (Mahambo, Vohitrambato, Sahamatevina and Manambotra Sud) randomly selected houses from the list of unsprayed houses identified from the spray record were used for the mosquito collection. At Vavatenina site, which was not sprayed, mosquitoes were collected from randomly selected houses. These mosquitoes were transported to the local test sites and used for insecticide resistance testing. The ages of mosquitoes used for the tests were not standardized. Mosquitoes of all blood digestion stages (unfed, fed, half gravid and gravid) were mixed and tested. The total number of mosquitoes obtained from the field collection determined the number of mosquitoes exposed to each insecticide.

#### Mosquito identification

Prior to testing, all adult mosquitoes were morphologically identified to species using standard identification keys [36]. All *Anopheles* specimens used for the susceptibility tests were labeled and stored individually in 1.5 ml Eppendorf tubes on silica gel and refrigerated at 4 °C before being shipped for molecular analysis. Subsets of the mosquitoes were further analyzed using a polymerase chain reaction (PCR) to confirm the species and assess mechanism of resistance.

### Susceptibility testing

Both WHO standard test kits for adult mosquitoes [37] and CDC bottle bioassays [38] were used to assess the susceptibility of mosquitoes to different insecticides approved by the WHO Pesticide Evaluation Scheme (WHOPES) for malaria vector control. Until 2012, only the WHO tube test was used for resistance testing in Madagascar. In that year, the CDC bottle bioassay was introduced to avoid the long lead times it requires to obtain WHO tube test kits and insecticide-impregnated papers from the Universiti Sains Malaysia. The CDC bottle bioassay also allows for assessing metabolic resistance mechanisms by pre-exposing mosquitoes to different synergists. National malaria control managers and in-country malaria partners asked if the results from the two test methods, even if not directly comparable, converge and lead to the same conclusion or classification of resistance of a mosquito population. To answer this question, the researchers collected data using both methods where possible. The following insecticides were used for the testing: DDT (4%, 100 μg/bottle), permethrin (0.75%, 21.51 μg/bottle), deltamethrin (0.05%, 12.5 μg/bottle), lambda-cyhalothrin (0.05%, 12.5 μg/bottle), alpha-cypermethrin (12.5 μg/bottle), bendiocarb (0.1%, 12.5 μg/bottle) and pirimiphos-methyl (0.25%, 20 μg/bottle). Diagnostic concentrations and time were used in both test methods according to standard protocols. Oil-impregnated papers and bottles coated with only acetone were used as controls for the WHO tube test and CDC bottle bioassay, respectively. Test kits, insecticide, and oil-impregnated papers were purchased from the Universiti Sains Malaysia for the WHO tube bioassay. CDC bottle bioassay kits, including technical grade insecticide, were obtained from CDC (Atlanta, USA). The insecticide-impregnated papers and insecticide stock solutions used for CDC bottle bioassays were stored at 4 °C in a refrigerator while at the central project office in Antananarivo. A cool box with frozen ice packs was used when transporting the papers from Antananarivo to the field test sites and when storing them in the temporary field offices. The temperature in the cool box was usually 4–8 °C, although on rare occasions it rose to 10 °C. Ice packs were frozen at the nearest health facility or hotel and changed as necessary to maintain a consistently low temperature in the cool box. In addition, the quality and efficacy of WHO insecticide-impregnated papers were checked and confirmed using susceptible strain mosquitoes after the fieldwork was complete. In the event that impregnated papers failed quality control tests after data collection, test results were removed from the data. The quality of each stock of insecticide used for CDC bottle bioassay was also tested using a susceptible colony in Antananarivo.

One hundred test female mosquitoes in four replicates (25 mosquitoes each) and 50 control female mosquitoes in two replicates were used in testing *An*. *gambiae* (*s.l.*). Owing to lower densities of the other two vectors in Madagascar, *An*. *mascarensis* and *An*. *funestus*, 18–65 and 30–75 female mosquitoes were used for tests, respectively. These tests were accompanied by 10–25 control female mosquitoes in one replicate as a negative control. Mortality was recorded after a 24 h holding period for WHO bioassays and at the end of the 30 min diagnostic time for CDC bottle bioassay except for DDT, which was 45 min. Mean test mortality was computed for each insecticide and method separately. Control mortalities were less than 5% in all the tests; therefore, use of a correction formula was not required. WHO criteria were used to classify vector susceptibility to each insecticide [37]. A mortality of 98% and greater was classified as full susceptibility, mortality below 90% was classified as resistance, and mortality of 90–97% was classified as suspected resistance that requires confirmation. A sample of dead and surviving *An*. *gambiae* (*s.l.*) specimens were randomly selected and used for molecular tests.

### Synergist bioassays

Pyrethroid and DDT resistance has been reported in *An*. *gambiae* (*s.l.*) in the absence of *kdr* from Madagascar [34], leaving metabolic resistance as the most likely resistance mechanism. To determine whether metabolic resistance mechanisms were indeed present, this study pre-exposed to two synergists non-blood-fed, two-to-five-day-old female *An*. *gambiae* (*s.l.*) mosquitoes reared from larvae and pupae collected from areas with suspected or confirmed resistance to pyrethroids. The synergists, piperonyl-butoxide (PBO) and S,S,S-tributyl phosphorotrithioate (DEF), are known to inhibit the activity of enzymes believed to detoxify public health insecticides. After a one-hour pre-exposure, the mosquitoes were exposed to diagnostic concentrations of various insecticides permethrin, deltamethrin, lambda-cyhalothrin and alpha-cypermethrin using CDC bioassay methods. The concentration of synergists used for the tests were prepared according to the CDC protocol [38] (100 μg/bottle for PBO and 125 μg/bottle for DEF) and used individually in all sites. Between 100 and 150 test mosquitoes (four to six replicates) were exposed to insecticide with or without pre-exposure to synergist (insecticide only or insecticide + synergist). Between 50 and 75 mosquitoes (two or three replicates) were used for the synergist bioassays as a control (exposed to neither synergist nor insecticide) simultaneously with the test mosquitoes. Fifty mosquitoes from each site were exposed to synergist only, except in Bekily where the synergist-insecticide bioassay was not accompanied by synergist-only due to a lack of mosquitoes. All the synergist-insecticide bioassays were conducted in 2016 independent of the susceptibility tests conducted in 2013/14 and 2015/16. Mortality was recorded after 30 min. Test results of each insecticide with and without mosquitoes’ pre-exposure to a synergist, and/or without pre-exposure to synergists were compared.

### Molecular identification of *An. gambiae* complex

All live samples, and a subset of dead samples, of the *An*. *gambiae* complex from DDT and pyrethroid resistance tests of 2015/2016 were subjected to molecular species identification using PCR as described by Scott et al. [39]. A total of 25–100 mosquitoes were sampled per test depending on the number mosquitoes that survived the insecticide exposure. Higher numbers of dead mosquitoes were randomly sampled from tests with higher numbers of mosquito survivors. Samples identified as *An*. *gambiae* after the species-specific assay were further amplified to differentiate between *An*. *coluzzii* and *An*. *gambiae* (*s.s.*), formerly called M and S molecular forms, respectively, by PCR according to Favia et al. [40].

### PCR detection of *kdr* (L1014F and L1014S) and *ace-1*^*R*^ mutations

The presence of L1014F and L1014S mutations was assessed from live and dead specimens of mosquitoes preserved following DDT and pyrethroid bioassay tests of 2015/16, using the standard PCR assays as described by Martinez-Torres et al. [41]. The samples were randomly selected. The PCR-RFLP diagnostic test was used to detect the *ace-1* ^*R*^ gene (G119S mutation) [42].

### Mosquito species and resistance

Association between mosquito species and frequency of resistance was assessed by combining results from the phenotypic resistance with molecular species identification. Species distribution in the dead and surviving subset was compared with the total population used for bioassays from each species to assess if there was any differential distribution of resistance between the two species.

### Data analysis

Fisher’s exact test was performed to determine if there was any significant difference in test mortality rates of *An*. *gambiae* (*s.l.*) mosquitoes with and without pre-exposure to synergists. In some sites, *An*. *gambiae* (*s.l.*) were pre-exposed to more than one synergist and mortality compared to tests without pre-exposure. In those cases, an overall test of equality was performed first, followed by multiple *post-hoc* comparisons with Bonferroni correction when the overall test results revealed significance. Fisher’s exact test also was used to determine any significant difference in the response to insecticides between *An*. *gambiae* and *An*. *arabiensis* where they occur in the same geographical area. The agreement between the WHO tube test and CDC bottle bioassay was compared using Kappa statistics [43]. Cohen’s kappa (κ) values were interpreted as poor (κ ≤ 0), slight (0 < κ ≤ 0.2), fair (0.2 < κ ≤ 0.4), moderate (0.4 < κ ≤ 0.6), substantial (0.6 < κ ≤ 0.8) and almost perfect agreement (0.8 < κ ≤ 1.0) [43]. Overall, mortality data were analyzed and interpreted according to the WHO 2013 protocol [37]. STATA 12 (Stata Corporation, USA) statistical package was used for this analysis.

## Results

### Profiling of *An*. *gambiae* (*s.l.*) phenotypic resistance to various insecticides

#### DDT

**Fig. 2:**
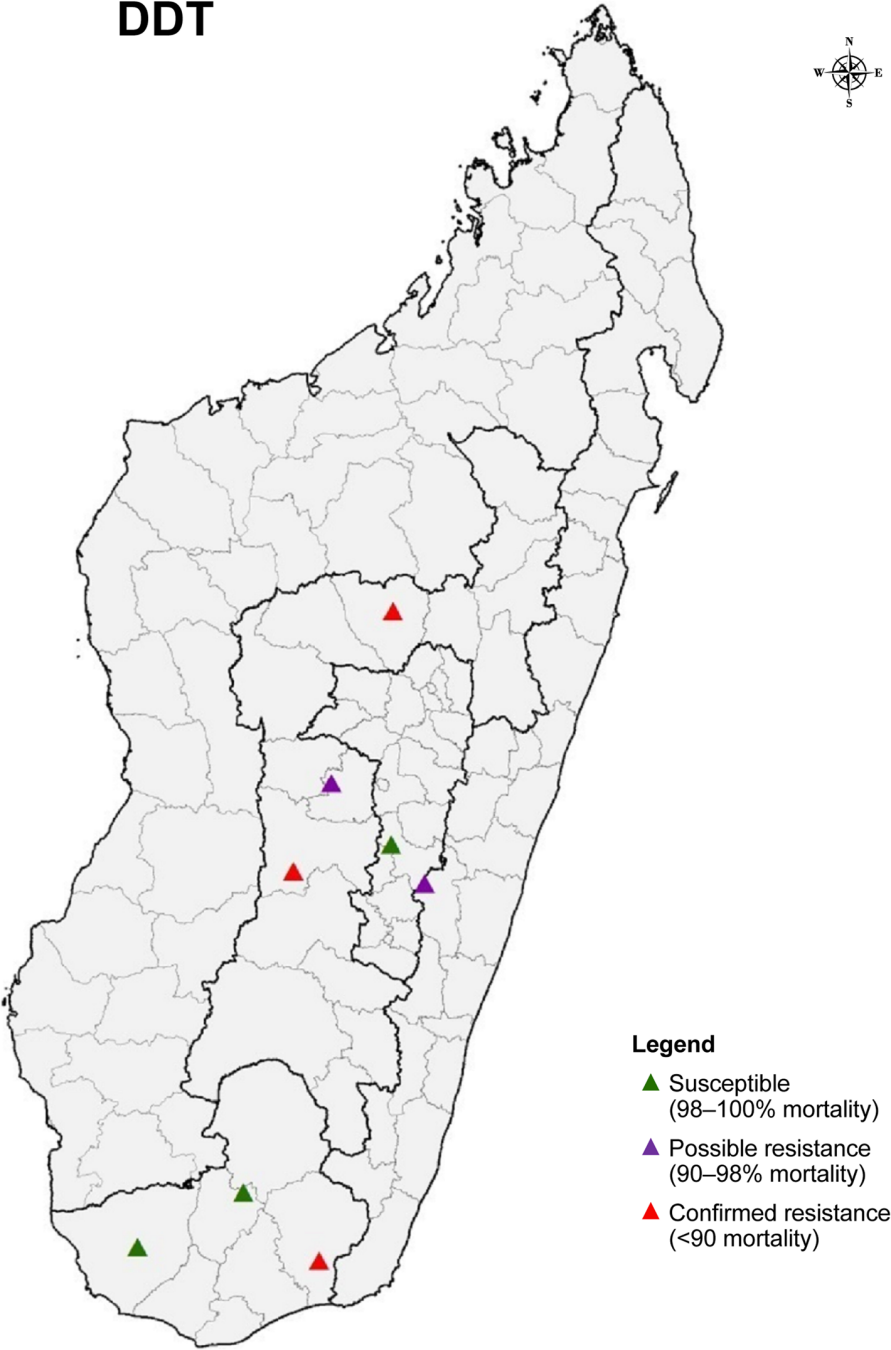
Distribution of DDT resistance of *An. gambiae* (*s.l.*) in Madagascar 2013–2014 monitored during one transmission period using CDC bottle bioassay

In 2013/2014, 800 female *An*. *gambiae* (*s.l.*) mosquitoes reared from larvae and pupae collected from eight study sites were exposed to DDT diagnostic dosages using the CDC bottle bioassays (100 per site) (Fig. 2, Additional file 1: Table S1 and Additional file 2: Figure S1). Resistance to DDT was noted in three sites: Amboasary (85% mortality), Kiangara (79% mortality) and Soavina (Ambatofinandrahana) (89% mortality). Results from two sites, Imerina Imaday and Bekily, indicated full susceptibility of the vector to DDT with test mortality of 100% in both sites. Suspected resistance to DDT was recorded in three sites, Manandory, Soavina (Betafo) and Ejeda, with a test mortality of 97% in all three sites (Fig. 2, Additional file 1: Table S1, Additional file 2: Figure S1).

#### Pyrethroids

**Fig. 3:**
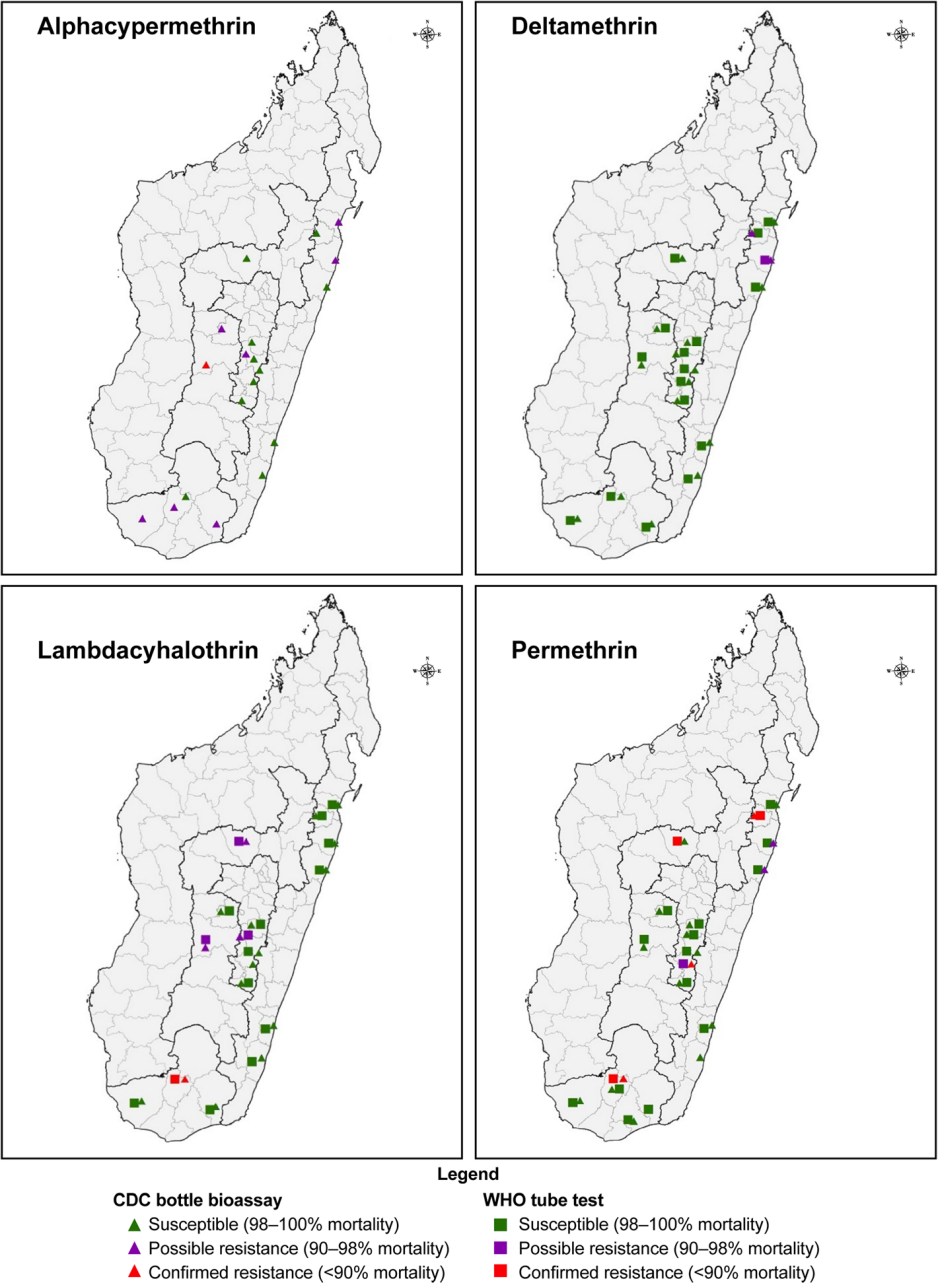
Distribution of pyrethroid resistance of *An. gambiae* (*s.l.*) in Madagascar tested 2013–2016 monitored in two rounds using both WHO and CDC bottle bioassays

In 2013/2014, using both WHO tube tests and CDC bottle assays, *An*. *gambiae* (*s.l.*) populations showed full susceptibility to deltamethrin in all eight study sites (mortality 98–100%). Lambda-cyhalothrin susceptibility was reported in six of the sites (mortality 98–100%), while populations from two sites were potentially resistant (mortality 93–97%). For these two insecticides, no difference in the classification of resistance status was observed between the two test methods. For permethrin, complete susceptibility was recorded in all test sites with the CDC bottle bioassay and in seven of the eight sites with the WHO tube test. Permethrin resistance was recorded with the WHO test in one site, Kiangara (mortality 80%). Only the CDC bottle bioassay was used to obtain exposure mortality point estimates of *An*. *gambiae* (*s.l.*) to alpha-cypermethrin. The vector was susceptible to alpha-cypermethrin (mortality 100%) in three sites, while populations from four sites were potentially resistant to the insecticide (mortality 95–97%). The population from Soavina (Ambatofinandrahana) was resistant to alpha-cypermethrin (mortality 89%) (Fig. 3, Additional file 1: Table S1, Additional file 3: Figure S2 and Additional file 4: Figure S3).

In 2015/2016, data from 11 sites showed full susceptibility of *An*. *gambiae* (*s.l.*) to alpha-cypermethrin in eight of the sites (mortality 99–100%) and potential resistance in three sites (mortality 91–97%).

The data showed full susceptibility to lambda-cyhalothrin in nine of the eleven sites. Populations from Bekily exhibited confirmed resistance and from Imerina Imady suspected resistance. For lambda-cyhalothrin, results from both test methods were in agreement in terms of the classification of resistance status for mosquitoes from the same site.

Conversely, differences were observed in the classification of resistance status of mosquito populations from the same study site when exposed to permethrin in three of the 11 sites (30% of the study sites) using the two test methods. In two sites, Vohitrambato and Ambodifaho, the *An*. *gambiae* (*s.l.*) were classified as susceptible based on the WHO tube test (mortality 100%), and suspected resistant based on the CDC bottle bioassay (mortality 95%). In Ankafina Tsarafidy, the mosquito population was classified as suspected resistant based on the WHO tube test (mortality 97%) and resistant based on the CDC bottle bioassay (mortality 68%). Results from the two test methods from eight sites were concordant and indicated *An*. *gambiae* (*s.l.*) populations from six sites were fully susceptible (mortality 99–100%), whereas populations from two sites were resistant to permethrin (Fig. 3, Additional file 1: Table S1, Additional file 3: Figure S2 and Additional file 4: Figure S3).

For deltamethrin, populations from nine of the 11 sites were fully susceptible based on results from both methods (mortality 98–100%). A discrepancy in resistance classification status was observed in Vavatenina, with point estimates of 98% from the WHO method and 96% from the CDC bottle bioassay. Suspected resistance to deltamethrin were noted in populations from Vohitrambato with 92% and 91% mortality with WHO tube and CDC bottle bioassays, respectively (Fig. 3, Additional file 1: Table S1, Additional file 3: Figure S2 and Additional file 4: Figure S3).

#### Pirimiphos-methyl and bendiocarb

**Fig. 4:**
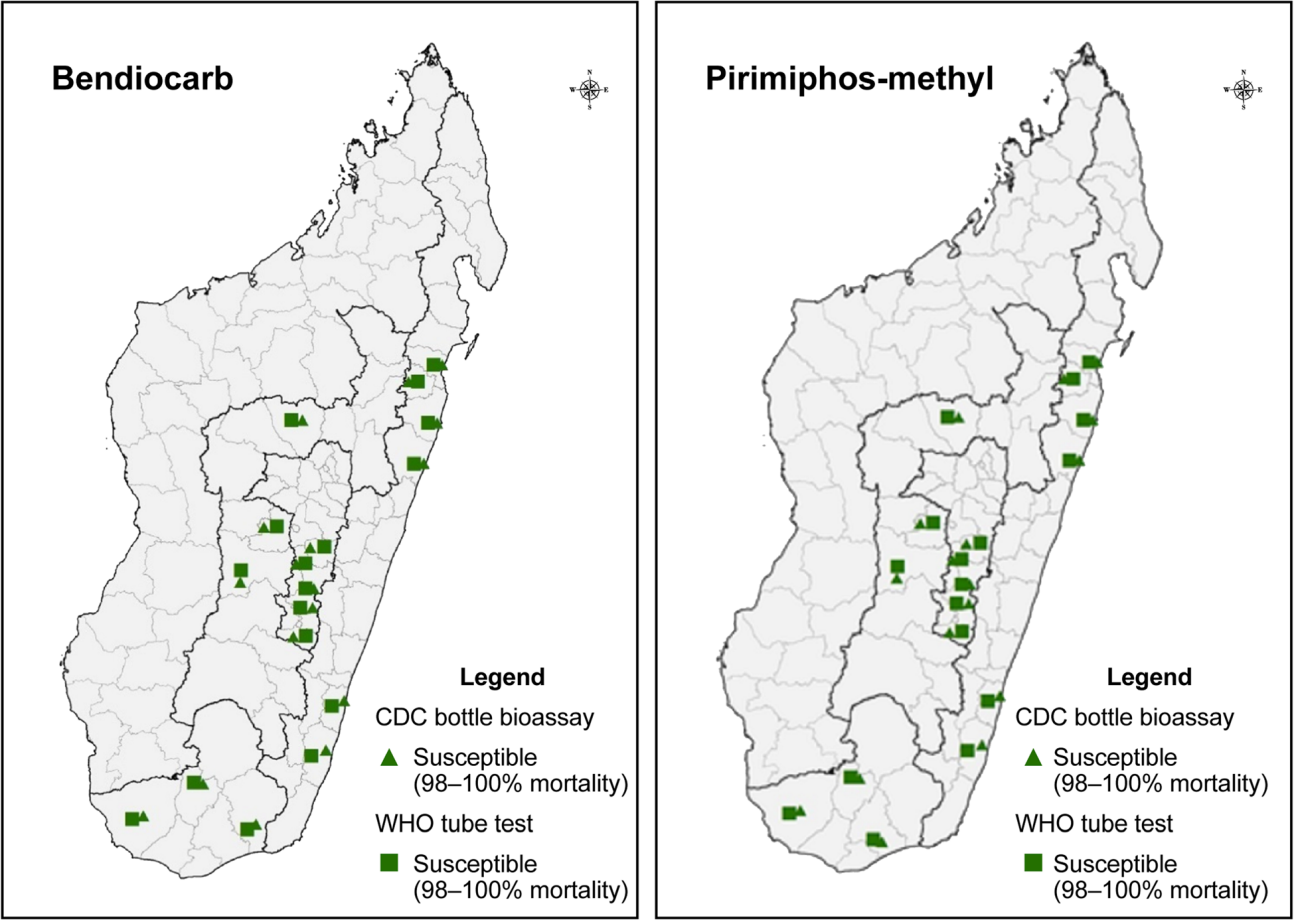
Distribution of bendiocarb and pirimiphos-methyl resistance of *An. gambiae* (*s.l.*) in Madagascar tested 2013–2016 monitored in two rounds using both WHO and CDC bottle bioassays

*Anopheles gambiae* (*s.l.*) populations from all study sites were found fully susceptible to pirimiphos-methyl and bendiocarb in both test years, based on results from test methods (Fig. 4, Additional file 1: Table S1).

### Profiling of *An. funestus* and *An. mascarensis*

CDC bottle bioassay results showed *An*. *funestus* and *An*. *mascarensis* were fully susceptible to pirimiphos-methyl, permethrin and deltamethrin (Table 2).

**Table 2:** *Anopheles funestus* and *An. mascarensis* insecticide susceptibility test results by site/village

District	Site / Village	Vector mosquito tested	Insecticide	% Mortality (*n*)	Resistance status
Farafangana	Manambotra Sud (SE)	*An*. *funestus*	Pirimiphos-methyl	100 (30)	S
		*An*. *funestus*	Deltamethrin	100 (75)	S
		*An*. *mascarensis*	Deltamethrin	100 (20)	S
Toamasina II	Vohitrambato (EC)	*An*. *funestus*	Pirimiphos-methyl	100 (35)	S
Fenerive Est	Mahambo (EC)	*An*. *mascarensis*	Pirimiphos-methyl	100 (18)	S
		*An*. *mascarensis*	Deltamethrin	100 (50)	S
Brickaville	Sahamatevina (EC)	*An*. *mascarensis*	Permethrin	100 (50)	S
Vavatenina	Vavatenina (EC)	*An*. *mascarensis*	Deltamethrin	100 (65)	S

*Abbreviations*: SE, south-east; EC, east coast; n, sample size; S, susceptible

### Synergist bioassays

The results of synergist bioassays are summarized in Table 3 and Additional file 5: Table S2. Pre-exposure to PBO and DEF either fully or partially restored susceptibility to the pyrethroid insecticides tested. Pre-exposure to PBO restored full susceptibility of *An*. *gambiae* (*s.l.*) to lambda-cyhalothrin, deltamethrin and alpha-cypermethrin in all six sites where tested. It also restored full susceptibility to permethrin in five of the sites; in Ambodifaho, it only partially restored susceptibility to permethrin (test mortality 97%). Pre-exposure to DEF completely eliminated *An*. *gambiae* (*s.l.*) resistance to deltamethrin and alpha-cypermethrin; it fully restored the mosquitoes’ susceptibility to permethrin in one of three sites and partially restored susceptibility to permethrin in the other two sites (mortality 91 and 92%, respectively).

**Table 3:** *Anopheles gambiae* (*s.l.*) insecticide susceptibility test results with and without pre-expsoure to synergists

Site (eco-epidemiological zone)	Insecticide tested	% Mortality (*n*)	Resistance status	*P*-value^a^
Imerina Imady (CHL)	L-cyhalothrin	94.67 (150)	PR	0.007***
	L-cyhalothrin + PBO	100 (150)	S	
Ankafina-Tsarafidy (CHL)	Permethrin	71.3 (150)	R	1
	Permethrin + PBO	100 (150)	S	< 0.001**
	Permethrin + DEF	91 (100)	PR	< 0.001**
Vavatenina (EC)	Permethrin	88.67 (150)	R	1
	Permethrin + PBO	100 (150)	S	0.001*
	Permethrin + DEF	92 (100)	PR	0.519
	Deltamethrin	94 (150)	PR	1
	Deltamethrin + PBO	100 (150)	S	0.003*
	Deltamethrin + DEF	100 (100)	S	0.012*
Bekily (SE)	Permethrin	75 (100)	R	< 0.001***
	Permethrin + PBO	100 (100)	S	
Ambodifaho (EC)	Permethrin	94 (150)	PR	0.256
	Permethrin + PBO	97.3 (150)	PR	
Vohitrambato (EC)	Permethrin	93.3 (150)	PR	0.002***
	Permethrin + PBO	100 (150)	S	
	Deltamethrin	92 (150)	PR	1
	Deltamethrin + PBO	100 (150)	S	< 0.001*
	Deltamethrin + DEF	100 (100)	S	0.002*
	a-cypermethrin	91 (100)	PR	0.003***
	a-cypermethrin + PBO	100 (100)	S	
Mahambo (EC)	Permethrin	83.3 (150)	R	1
	Permethrin + PBO	100 (150)	S	< 0.001*
	Permethrin + DEF	100 (100)	S	< 0.001*
	a-cypermethrin	90.67 (150)	PR	1
	a-cypermethrin + PBO	100 (150)	S	< 0.001*
	a-cypermethrin + DEF	100 (100)	S	0.001*

^a^Reported *P*-values of comparative analysis of synergist exposure for each sampling location

*Statistically significant at *P* < 0.0167

**Statistically significant at *P* < 0.0125

***Statistically significant at *P* < 0.05

*Abbreviations*: CHL, central highlands; EC, east coast; SE, south-east; n, sample size; PR, possible resistance; S, susceptible; R, confirmed resistance

### Mosquito species, *kdr* L1014 and G119S allelic and genotype frequencies

A total of 1006 *An*. *gambiae* (*s.l.*) mosquitoes preserved after insecticide resistance testing in the eight sites were analyzed for species identification and presence of *kdr* mutation; 986 samples were amplified. A more limited sample of mosquito from three sites was also genotyped for detection of the G119S mutation (*n* = 248). All mosquito samples analyzed from the three sites in the CHL (Imerina Imady, Milamaina and Vohimarina) were found to be *An*. *arabiensis* (*n* = 465). Specimens from the east coast and south sub-desert were mainly *An*. *gambiae* and *An*. *arabiensis*. Fourteen *An*. *coluzzii* and one *An. merus* also were identified from samples analyzed from Vavatenina and Bekily, respectively. No L1014F or L1014S mutations were found among the 986 specimens analyzed for *kdr*. All 248 mosquitoes analyzed for *ace-1* ^*R*^ were negative for G119S mutations (Table 4).

**Table 4:** Molecular analysis results of mosquitoes specimens from three different eco-epidemiological zones in Madagascar

Study site (eco-epidemiological zone)	*An. gambiae* (*s.s.*)	*An. arabiensis*	*An. coluzzi*	*An. merus*	Total
	*n* (%)^a^	*n* (%)	*n* (%)	*n* (%)	*n* (%)
Imerina Imady (CHL)	0	150 (100)	0	0	150
Vohitrambato (EC)	75 (93.0)	6 (7.0)	0	0	81
Vavatenina (EC)	4 (11.5)	20 (52.6)	14 (36.8)	0	38
Bekily (SE)	94 (66.0)	47 (33.0)	0	1 (0.7)	142
Ambodifaho (EC)	46 (92.0)	4 (8.0)	0	0	50
Mahambo (EC)	144 (69.0)	66 (31.0)	0	0	210
Milamaina (CHL)	0	216 (100)	0	0	216
Vohimarina (CHL)	0	99 (100)	0	0	99
Total	363 (36.8)	608 (61.7)	14 (1.4)	1 (0.1)	986

^a^Number and percentage of members of *Anopheles gambiae* complex after molecular species identification in each of the study sites

*Abbreviations*: CHL, central highlands; EC, east coast; SE, south-east

### Mosquito species and resistance

The results of association between mosquito species and frequency of resistance are summarized in Table 5. In Bekily and Mahambo, *An*. *gambiae* and *An*. *arabiensis* live in the same geographical areas. In Bekily, the mortality of *An*. *arabiensis* was significantly lower than that of *An*. *gambiae* when exposed to deltamethrin and permethrin (*P* < 0.001). Conversely, in Mahambo, with WHO tube bioassays, the mortality rate of *An*. *arabiensis* was higher than that of *An*. *gambiae* when exposed to permethrin (*P* < 0.001). There was no significant difference in the mortality rate when *An*. *arabiensis* and *An*. *gambiae* populations from Mahambo were exposed to DDT (*P* = 0.99) and alpha-cypermethrin (*P* = 0.018).

**Table 5:** Species distribution in dead and alive *Anopheles gambiae (s.l.)* mosquitoes after susceptibility tests

Site and insecticide tested (*n*)	*An. arabiensis*	*An. gambiae* (*s.s.*)	Total assayed	*P*-value
Bekily (CDC bottle bioassay)				
Deltamethrin survivors	10	0	10	< 0.001*
Deltamethrin dead	2	34	36	
Deltamethrin total	12	34	46	
Permethrin survivors	14	3	17	< 0.001*
Permethrin dead	21	57	78	
Permethrin total	35	60	95	
Mahambo (CDC bottle bioassay )				
Alpha-cypermethrin survivors	6	3	9	0.018*
Alpha-cypermethrin dead	7	25	32	
Alpha-cypermethrin total	13	28	41	
DDT survivors	3	3	6	0.99
DDT dead	20	22	42	
DDT total	23	25	48	
Mahambo (WHO tube bioassay)				
Permethrin survivors	0	42	42	< 0.001*
Permethrin dead	30	49	79	
Permethrin total	30	91	121	

*Statistically significant at *P* < 0.05

## Discussion

This study’s main objective was to conduct and report on the distribution and frequency of insecticide resistance in the three known malaria vectors in Madagascar, in order to better guide insecticide-based vector control. The study covered three of the country’s five eco-epidemiological zones for *An*. *gambiae* (*s.l.*) and two for *An*. *funestus* and *An*. *mascarensis*.

Consistent with earlier studies [33, 34], susceptibility test results from this study indicate a high prevalence of *An*. *gambiae* (*s.l.*) resistance to DDT and permethrin relative to the other insecticides tested. Resistance to alpha-cypermethrin and lambda-cyhalothrin was observed in a few sites at low frequencies; no confirmed resistance to deltamethrin was recorded. Rakotondrainble et al. [33] reported no phenotypic resistance in *An. gambiae* (*s.l.*) in the CHL to deltamethrin and lambda-cyhalothrin, but they detected resistance to permethrin in one site, Alasora. Ratovonjato et al. [34] detected possible resistance to alpha-cypermethrin in two sites in the CHL. This study indicated suspected resistance to alpha-cypermethrin in two of seven tests, with mortality of 97% in Soavina (Betafo) and 95% in Imerina Imady, and full susceptibility in five of the sites. Hence, there is no evidence to support an increase in frequency of resistance to pyrethroids over the past decade (1996–2015); the frequency of resistance to DDT and pyrethroids appears stable.

As noted above, IRS was done consistently for malaria vector control in Madagascar from 1949 [29] until 1979, and was re-introduced in 1993 [30]; in 2008, the Ministry of Health began scaling up ITN distribution, with coverage quickly reaching 79% of households [31]. Despite the consistent use of these two vector control methods with insecticides that target the sodium channels of insect vectors that can confer resistance to both pyrethroids and DDT, molecular analyses by this study and an earlier one [34] observed no detectable level of *kdr* mutation. One explanation could be that Madagascar is geographically isolated, making the introduction of vector populations carrying *kdr* alleles associated with resistance unlikely. It also is possible that local selection pressure from the insecticides might not be enough to maintain naturally occurring, low-frequency mutations in the mosquito population, particularly if carrying the resistant genes comes with some fitness cost. The absence of *kdr* mutations in turn might totally, or partially, explain why the emergence and spread of DDT and pyrethroid phenotypic resistance in Madagascar has been slower than in most African countries, where there is easier flow of resistant genes between the mosquito populations of neighboring territories. Insecticide selection pressure from agriculture might also be different from mainland Africa.

With the exception of two sites, Imerina Imady and Bekily, the 2015/2016 resistance tests were conducted in sites different from sites used in 2013/2014; while this provides more representative data, it makes it hard to make comparisons between years. For example, in 2013/2014, *An*. *gambiae* (*s.l.*) from Imerina Imady was fully susceptible (mortality ≥ 98%) to alpha-cypermethrin, bendiocarb, DDT, deltamethrin, lambda-cyhalothrin, permethrin and pirimiphos-methyl, whereas in 2015/2016, possible resistance to the pyrethroids alpha-cypermethrin and lambda-cyhalothrin was recorded. The different results may have been due to differences in larval collection locations, timing of collections/testing, species composition or emergence over time of resistant individuals.

With regard to the distribution of members of the *An*. *gambiae* complex, only *An*. *arabiensis* was found in CHL. *Anopheles gambiae* (*s.s.*) was predominant in all east coast study sites except Vavatenina, where *An*. *arabiensis* was dominant. *Anopheles gambiae* (*s.s.*) was more prevalent in the south sub-desert. These members of the *An*. *gambiae* complex may respond differently to the various insecticides. Differences in resistance status between *An*. *gambiae* complex members have been seen elsewhere. Ochomo et al. [44] reported high *kdr* frequency in *An*. *arabiensis* (75.9%) compared with *An*. *gambiae* (*s.s.*) (11.1%) in the Bungoma district of Kenya, where the two species live in sympatry, as well as differences in phenotypic resistance. Conversely, in Tanzania, high phenotypic resistance to pyrethroids and DDT and high *kdr* frequencies were seen in *An*. *gambiae* (*s.s.*), while *An*. *arabiensis* populations were fully susceptible to all pyrethroids tested except permethrin with no *kdr* mutations [4].

In this study, no significant difference in resistance status was noted between *An*. *arabiensis* and *An*. *gambiae* in Mahambo district (east coast) when tested against alpha-cypermethrin. However, in Bekily (southern Madagascar), where *An*. *arabiensis* and *An*. *gambiae* larvae were collected from the same breeding sites, a significantly higher proportion of *An*. *arabiensis* survivors was observed as compared to *An*. *gambiae* when tested against deltamethrin and permethrin (Fisher’s exact test, *P* < 0.001). Olyset nets (impregnated with permethrin) and PermaNet (impregnated with deltamethrin) were deployed to Bekily in 2010 and 2015, respectively. A previous study in Kenya reported a correlation between ITNs scale-up and increased prevalence of insecticide resistance [45]. It is not clear whether selection pressure from ITNs has played a role in the differential response in the two species observed in this study. However, the bioassay results showed that three rounds of IRS application with a carbamate (bendiocarb WP 80) between 2010 and 2013 and one round of an organophosphate (pirimiphos-methyl 300 CS) in 2014 has not selected a detectable level of resistance to these two classes of insecticides; the vector was fully susceptible to both insecticides. Assessing the extent of use and the contribution of agricultural insecticides to the observed malaria vector resistance in Madagascar was not part of the scope of this study, but it should be included in future studies to better understand the evolution of resistance and to design evidence-based mitigation strategies.

Pre-exposing mosquitoes to synergists PBO, an inhibitor of oxidases, and DEF, an inhibitor of non-specific esterases (NSEs), prior to the bioassay tests restored at least partial susceptibility of *An*. *gambiae* (*s.l.*) to pyrethroids in CDC bottle bioassays, suggesting the two categories of detoxifying enzymes (NSE, and cytochrome P450 monoxygenases) have played an important role in the evolution and shaping of insecticide resistance in Madagascar. With the exception of one site, where only partial restoration of susceptibility to permethrin was recorded, complete pyrethroid resistance suppression was achieved for insecticides tested with pre-exposure to PBO. With pre-exposure to DEF, only partial suppression of resistance to permethrin was achieved in two of three vector populations. Nevertheless, pre-exposure to PBO or DEF fully synergized resistance to deltamethrin and alpha-cypermethrin. Inhibiting one of these two enzymes appeared sufficient to restore the effectiveness of these two insecticides to which resistance had been suspected (not confirmed). However, inhibition of NSE alone was not always sufficient to fully restore susceptibility to permethrin in areas where resistance to this insecticide had been confirmed, indicating the dominant role of mixed function oxidases (MFOs) in *An*. *gambiae* (*s.l.*) resistance to permethrin. Further investigation is needed to learn if MFOs also play crucial role in vector resistance to deltamethrin and alpha-cypermethrin where confirmed resistance to these insecticides exists.

Absence of *kdr* mutations coupled with partial or complete restoration of susceptibility to pyrethroids with vector pre-exposure to synergists indicates the critical role that metabolic resistance mechanisms play in shaping phenotypic resistance in Madagascar. Further biochemical analysis is needed to determine the specific gene or genes responsible for vector survival. The absence of cross-resistance between DDT and pyrethroids is further evidence of the absence of the role of *kdr* in determining vector resistance to these insecticides. *Kdr*-free pyrethroid resistance in *An*. *arabiensis* attributed to metabolic resistance was detected in Chad [46]. Similarly, Verhaeghen et al. [47] reported the absence of *kdr* in several vectors of malaria in the Mekong region resistant to DDT and pyrethroids with elevated detoxifying enzymes. Unlike *kdr*, all insects are reported to have enzyme-based adaptive mechanisms that would help them metabolize xenobiotics including insecticides [48]. This might be the most likely reason why resistance due to metabolic mechanism was observed in Madagascar though the country is geographically isolated and the gene flow from other malaria endemic countries is restricted.

Results from both WHO tube and CDC bottle bioassays demonstrated the full susceptibility of all three malaria vectors in the country to bendiocarb (carbamate) and pirimiphos-methyl (organophosphate) insecticides. The G119S mutation associated with resistance to carbamates and organophosphates was also not detected. This is encouraging because it means Madagascar has several effective insecticides options to choose from when planning insecticide-based vector control, unlike most other sub-Saharan Africa countries. The other secondary vectors, *An*. *funestus* and *An*. *mascarensis*, were fully susceptible to pyrethroids and organophosphates.

As noted above, the WHO tube bioassay was the only standard resistance testing procedure used in Madagascar until the CDC bottle bioassay method was deployed in 2012. There are some differences in the diagnostic dosages and what the two techniques measure. The WHO assay diagnostic dosage is twice the lowest concentration that produces 100% mortality after a 60 min exposure and a holding period of 24 h on a susceptible strain/population. The CDC bottle bioassay uses an insecticide that kills all (100%) susceptible mosquitoes within a given diagnostic time (45 min for DDT and 30 min for the other public health insecticides).

Of 95 tests (*n* = 9500) conducted using both methods, 90 concordant results (94.73%) and five discordant ones (5.26%) were observed in resistance classification. When the data were broken down by insecticide, all the test results from the two test methods converged and no discrepancy was observed in bendiocarb (κ = 1), pirimiphos-methyl (κ = 1) and lambda-cyhalothrin (κ = 0.70). The five discordant results all came from two insecticides, four from permethrin and one from deltamethrin. Further investigation is needed to learn why test results for permethrin differ between the two test methods (κ = 0.45). From our limited observation, the results from the two test methods seemed to vary as the frequency of resistance increased. The discordant results observed in deltamethrin, with mortality of 98% and 96% in one site, was not beyond variation that could be expected due to chance alone (κ = 0.77). Aïzoun et al. [49] reported comparable results from both assays, which is consistent with our results. The monitoring, separately or in parallel, for bendiocarb, pirimiphos-methyl, deltamethrin and lambda-cyhalothrin and give similar results in Madagascar where there is no or very infrequent resistance to these insecticides.

The resistance status of vectors in Madagascar shows the potential for malaria vector control to be effective there. The absence of widespread vector resistance to insecticides other than DDT and permethrin will enable the vector control program to develop a preemptive and evidence-based insecticide use strategy aimed at preventing the development of widespread resistance and preserving the ongoing impact of control efforts.

Though it yielded important findings, this study had limitations. Wild-caught female adult *An*. *funestus* and *An. mascarensis* (a mix of old and young age) mosquitoes were directly used for testing. The results indicated full susceptibility of the vectors to the insecticides tested. Previous studies have reported a correlation between mosquito age and mortality (increased mortality with increasing age) in *An*. *gambiae* (*s.l.*) [50] and *An*. *funestus* [51]. The absence of resistance in these two vector species, therefore, may be explained by either full susceptibility of the vector to the insecticides tested or by resistance possibly masked by the use of mixed mosquito ages. Among the mosquitoes exposed to insecticides, none survived. Hence, the first explanation appears more plausible as the use of mixed-age groups may not totally abolish resistance if it exists in the population.

Another limitation is that not all of the susceptibility tests were accompanied by a test of a susceptible mosquito colony as a reference (positive control) in the field due to the logistical challenges of safely transporting susceptible mosquitoes to the remote test sites and lack of an adequate number of mosquitoes in the insectary. However, the quality and efficacy of the WHO impregnated papers and insecticide stock solution used for CDC bottle bioassay were checked and confirmed for the test results reported in this paper.

In one of the study areas, Bekily, PBO-only was not included as a control during the synergist-insecticide testing due to an inadequate number of mosquitoes. Some of the mosquito mortality observed in Bekily could therefore be due to the synergist alone. However, there was no mosquito mortality attributable to PBO from the other sites tested, and therefore it is unlikely that PBO alone was the cause of any mortality in Bekily.

Finally, biochemical analysis was not performed to support phenotypic resistance data obtained with and/or without pre-exposing mosquitoes to the synergists. This was due to lack of a facility in country. Further study is needed to determine the specific genes responsible for metabolic resistance detected in Madagascar.

Despite these limitations, the strengths of this study are the inclusion of three known malaria vectors, the number and geographical distribution of sentinel sites (18 in three of the five eco-epidemiological zones) and the number and types of insecticides tested. The information gathered is substantial and will contribute to informing country malaria control policies.

## Conclusions

The limited insecticide resistance observed in Madagascar’s malaria vector populations presents an opportunity for the country’s vector control program to maintain a range of viable insecticides for use in the ongoing mission to eliminate malaria. This situation provides a unique opportunity to implement preemptive insecticide rotation to preserve existing vector control tools and creates space for continued cost-effective vector control programming. If implemented successfully, a strong resistance management plan could provide a working model for other regions that are currently limited by widespread resistance to available insecticides, with the expectation that novel products will be available in the near future.

## Additional files

Additional file 1: Table S1. *Anopheles gambiae* (*s.l.*) WHO tube and CDC bottle bioassay results by site/village. Sample size is in parentheses (*N*). (DOCX 48 kb)

Additional file 2: Figure S1. Mortality rates of *An.gambiae* (*s.l*.) field populations exposed to DDT diagnostic dosage between 2013 and 2014 in eight sentinel sites in Madagascar. (PPTX 42 kb)

Additional file 3: Figure S2. Mortality rates of *An.gambiae* (*s.l*.) field populations exposed to alpha-cypermethrin, permethrin, deltamethrin and lambda-cyhalothrin diagnostic dosages between 2013 and 2014 in nine sentinel sites in Madagascar (PPTX 48 kb)

Additional file 4: Figure S3. Mortality rates of *An.gambiae* (*s.l*.) field populations exposed to alpha-cypermethrin, permethrin, deltamethrin and lambda-cyhalothrin diagnostic dosages between 2015 and 2016 in eleven sentinel sites in Madagascar (PPTX 48 kb)

Additional file 5: Table S2. *Anopheles gambiae* (*s.l.*) insecticide susceptibility test results with and without pre-exposure to synergists by site/village using CDC bottle bioassays (DOCX 18 kb)

## Abbreviations

*ace-1*^*R*^: Acetylcholinesterase resistance
AIRS: Africa Indoor Residual Spraying
CDC: United States Centers for Disease Control and Prevention
CHL: Central highlands
DDT: Dichlorodiphenyltrichloroethane
DEF: S,S,S-tributyl phosphorotrithioate
EC: East Coast
IRS: Indoor residual spraying
ITN: Insecticide-treated bed net
*Kdr*: Knock-down resistance
MFOs: Mixed Function Oxidases
NSE: Non-Specific Esterase
OP: Organophosphate
PBO: Piperonyl-butoxide
PMI: US President’s Malaria Initiative
PY: Pyrethroid
SE: South East
SR: Suspected resistance
SUS: Susceptible
USAID: United States Agency for International Development
WHO: World Health Organization
