# Supplementary material for: Insecticide resistance status of three malaria vectors, Anopheles gambiae (s.l.), An. funestus and An. mascarensis, from the south, central and east coasts of Madagascar
Source: Parasit Vectors. 2017 Aug 23;10:396. doi: 10.1186/s13071-017-2336-9 (PMC5569519; doi:10.1186/s13071-017-2336-9)
Supplement: Supplementary file 1 — Anopheles gambiae (s.l.) WHO tube and CDC bottle bioassay results by site/village. Sample size is in parentheses (N). (DOCX 48 kb) [file 13071_2017_2336_MOESM1_ESM.docx]

**Additional file 1: Table S1** *Anopheles gambiae* (*s.l.*) WHO tube and CDC bottle bioassay results by site/village. Sample size is in parentheses (N).

| Year | District | Site / village | Insecticide tested | WHO tube test | | CDC bottle bioassay | | Temperature | | %RH | | Control | |
| --- | --- | --- | --- | --- | --- | --- | --- | --- | --- | --- | --- | --- | --- |
|  |  |  |  | % Mortality (N) | Resistance status | % Mortality (N) | Resistance status | WHO tube test | CDC bottle bioassay | WHO tube test | CDC bottle bioassay | WHO tube test | CDC bottle bioassay |
|  |  |  |  |  |  |  |  |  |  |  |  | % mortality (N) | % mortality (N) |
| 2013/2014 | Ambositra (CHL) | Imerina Imady | a-cypermethrin | 0 | ND | 100(100) | S | ND* | 24 | ND | 80 | ND | 0% (50) |
|  |  |  | bendiocarb | 98(100) | S | 99(100) | S | 24 | 24 | 79 | 80 | 0% (50) | 0% (50) |
|  |  |  | DDT | RD* | RD | 100(100) | S | RD | 25 | RD | 75 | RD | 0% (50) |
|  |  |  | deltamethrin | 99(100) | S | 100(100) | S | 25 | 25 | 80 | 75 | 0% (50) | 0% (50) |
|  |  |  | l-cyhalothrin | 100(100) | S | 99(100) | S | 25 | 25 | 80 | 75 | 0% (50) | 0% (50) |
|  |  |  | permethrin | 99(100) | S | 99(100) | S | 24 | 24 | 81 | 81 | 0% (50) | 0% (50) |
|  |  |  | p-methyl | 100(100) | S | 100(100) | S | 24 | 24 | 81 | 81 | 0% (50) | 0% (50) |
|  | Ambohimahasoa(CHL) | Manandroy | a-cypermethrin | 0 | ND | 100(100) | S | ND | 23 | ND | 75 | ND | 0% (50) |
|  |  |  | bendiocarb | 100 (100) | S | 100(100) | S | 23 | 23 | 75 | 75 | 0% (50) | 0% (50) |
|  |  |  | DDT | RD | RD | 97(100) | P | RD | 23 | RD | 75 | RD | 0% (50) |
|  |  |  | deltamethrin | 100(100) | S | 100(100) | S | 23.5 | 23.5 | 80 | 80 | 0% (50) | 0% (50) |
|  |  |  | l-cyhalothrin | 100(100) | S | 100(100) | S | 23.5 | 23.5 | 80 | 80 | 0% (50) | 0% (50) |
|  |  |  | permethrin | 100(100) | S | 100(100) | S | 23.5 | 23.5 | 80 | 80 | 0% (50) | 0% (50) |
|  |  |  | p-methyl | 100(100) | S | 100(100) | S | 23 | 23 | 72 | 72 | 0% (50) | 0% (50) |
|  | Betafo(fringe) | Soavina | a-cypermethrin | 0 | ND | 97(100) | P | ND | 23 | ND | 73 | ND | 0% (50) |
|  |  |  | bendiocarb | 100(100) | S | 100(100) | S | 23 | 23 | 73 | 73 | 0% (50) | 0% (50) |
|  |  |  | deltamethrin | 100(100) | S | 98(100) | S | 23 | 23 | 73 | 73 | 0% (50) | 0% (50) |
|  |  |  | DDT | RD | RD | 97(100) | P | RD | 23 | RD | 73 | RD | 0% (50) |
|  |  |  | l-cyhalothrin | 100(100) | S | 100(100) | S | 23 | 23 | 73 | 73 | 0% (50) | 0% (50) |
|  |  |  | permethrin | 100(100) | S | 100(100) | S | 23 | 23 | 73 | 73 | 0% (50) | 0% (50) |
|  |  |  | p-methyl | 100(100) | S | 100(100) | S | 23 | 23 | 73 | 73 | 0% (50) | 0% (50) |
|  | Ambatofinandrahana( fringe) | Soavina | a-cypermethrin | 0 | ND | 89 (100) | R | ND | 27 | ND | 72 | ND | 0% (50) |
|  |  |  | bendiocarb | 100(100) | S | 100(100) | S | 26 | 27 | 71 | 72 | 0% (50) | 0% (50) |
|  |  |  | DDT | RD | RD | 89(100) | R | RD | 27 | RD | 72 | RD | 0% (50) |
|  |  |  | deltamethrin | 100(100) | S | 99(100) | S | 26 | 27 | 71 | 72 | 0% (50) | 0% (50) |
|  |  |  | l-cyhalothrin | 97(100) | P | 95(100) | P | 25 | 27 | 71 | 72 | 0% (50) | 0% (50) |
|  |  |  | permethrin | 99(100) | S | 99(100) | S | 25 | 27 | 71 | 72 | 0% (50) | 0% (50) |
|  |  |  | p-methyl | 100(100) | S | 100(100) | S | 25 | 26.5 | 71 | 72 | 0% (50) | 0% (50) |
|  | Ankazobe ( fringe) | Kiangara | a-cypermethrin | 0 | ND | 100(100) | S | ND | 25 | ND | 80 | ND | 0% (50) |
|  |  |  | bendiocarb | 100(100) | S | 100(100) | S | 25 | 25 | 80 | 75 | 0% (50) | 0% (50) |
|  |  |  | DDT | RD | RD | 79(100) | R |  | 25 |  | 75 | RD | 0% (50) |
|  |  |  | deltamethrin | 98(100) | S | 99(100) | S | 25 | 25 | 80 | 75 | 0% (50) | 0% (50) |
|  |  |  | l-cyhalothrin | 96(100) | P | 93(100) | P | 24 | 24 | 81 | 81 | 0% (50) | 0% (50) |
|  |  |  | permethrin | 80(100) | R | 98(100) | S | 24 | 24 | 81 | 81 | 0% (50) | 0% (50) |
|  |  |  | p-methyl | 100(100) | S | 100(100) | S | 24 | 24 | 81 | 81 | 0% (50) | 0% (50) |
|  | Ambovombe (south sub-desert) | Ambovombe | P-methyl | 100(100) | S | 100(100) | S | 25 | 25 | 71 | 71 | 0% (50) | 0% (50) |
|  |  |  | permethrin | 0 | ND | 99(100) | S | ND | 25 | ND | 71 | ND | 0% (50) |
|  | Ampanihy(south sub-desert) | Ejeda | a-cypermethrin | 0 | ND | 95(100) | P | ND | 27 | ND | 70 | ND | 0% (50) |
|  |  |  | bendiocarb | 100(100) | S | 100(100) | S | 27 | 27 | 70 | 70 | 0% (50) | 0% (50) |
|  |  |  | DDT | RD | RD | 97(100) | P | RD | 27 | RD | 70 | RD | 0% (50) |
|  |  |  | deltamethrin | 98(100) | S | 98(100) | S | 27 | 27 | 70 | 70 | 0% (50) | 0% (50) |
|  |  |  | l-cyhalothrin | 99(100) | S | 100(100) | S | 26.5 | 26.5 | 75 | 75 | 0% (50) | 0% (50) |
|  |  |  | permethrin | 98(100) | S | 99(100) | S | 26.5 | 26.5 | 75 | 75 | 0% (50) | 0% (50) |
|  |  |  | p-methyl | 100(100) | S | 100(100) | S | 26.5 | 26.5 | 75 | 75 | 0% (50) | 0% (50) |
|  | Bekily(south sub-desert) | Bekily | a-cypermethrin | 0 | ND | 95(100) | P | ND | 27 | ND | 70 | ND | 0% (50) |
|  |  |  | bendiocarb | 98(100) | S | 100(100) | S | 27 | 27 | 70 | 70 | 0% (50) | 0% (50) |
|  |  |  | DDT | RD | RD | 100(100) | S | RD | 27 | RD | 70 | RD | 0% (50) |
|  |  |  | deltamethrin | 98(100) | S | 100(100) | S | 27 | 27 | 70 | 70 | 0% (50) | 0% (50) |
|  |  |  | l-cyhalothrin | 100(100) | S | 100(100) | S | 27 | 27 | 70 | 70 | 0% (50) | 0% (50) |
|  |  |  | permethrin | 99(100) | S | 99(100) | S | 27 | 27 | 70 | 70 | 0% (50) | 0% (50) |
|  |  |  | p-methyl | 100(100) | S | 100(100) | S | 27 | 27 | 70 | 70 | 0% (50) | 0% (50) |
|  | Amboasary(south sub-desert) | Amboasary | a-cypermethrin | 0 | ND | 96(100) | P | ND | 27 | ND | 70 | ND | 0% (50) |
|  |  |  | bendiocarb | 100(100) | S | 100(100) | S | 24 | 24 | 70 | 70 | 0% (50) | 0% (50) |
|  |  |  | DDT | RD | RD | 85(100) | R | RD | 24 | RD | 75 | RD | 0% (50) |
|  |  |  | deltamethrin | 100(100) | S | 99(100) | S | 24 | 24 | 70 | 75 | 0% (50) | 0% (50) |
|  |  |  | l-cyhalothrin | 98(100) | S | 99(100) | S | 27 | 27 | 75 | 75 | 0% (50) | 0% (50) |
|  |  |  | permethrin | 98(100) | S | 0 | ND | 27 | 27 | 75 | 75 | 0% (50) | ND |
| 2015/2016 | Fandriana (CHL) | Milamaina | a-cypermethrin | 0 | ND | 100(100) | S | ND | 25 | ND | 70 | ND | 0% (50) |
|  |  |  | bendiocarb | 100(100) | s | 100(100) | S | 26 | 26 | 75 | 70 | 0% (50) | 0% (50) |
|  |  |  | deltamethrin | 100(100) | S | 100(100) | S | 25 | 25 | 75 | 70 | 0% (50) | 0% (50) |
|  |  |  | l-cyhalothrin | 100(100) | S | 100(100) | S | 23 | 23 | 75 | 70 | 0% (50) | 0% (50) |
|  |  |  | permethrin | 100(100) | S | 100(100) | S | 23 | 23 | 75 | 70 | 0% (50) | 0% (50) |
|  |  |  | p-methyl | 100(100) | S | 100(100) | S | 24 | 24 | 75 | 70 | 0% (50) | 0% (50) |
|  | Ambositra(CHL) | Imerina Imady | a-cypermethrin | 0 | ND | 95(100) | P | ND | 26 | ND | 88 | ND | 0% (50) |
|  |  |  | bendiocarb | 100(100) | S | 100(100) | S | 26 | 26 | 83 | 88 | 0% (50) | 0% (50) |
|  |  |  | deltamethrin | 100(100) | S | 99(100) | S | 25.5 | 26 | 89 | 84 | 0% (50) | 0% (50) |
|  |  |  | l-cyhalothrin | 96(100) | P | 97(100) | P | 24.5 | 25.5 | 86.5 | 85 | 0% (50) | 0% (50) |
|  |  |  | permethrin | 99(100) | S | 100(100) | S | 25.5 | 26.5 | 89 | 84 | 0% (50) | 0% (50) |
|  |  |  | p-methyl | 100(100) | S | 100(100) | S | 24.5 | 26.5 | 86.5 | 87 | 0% (50) | 0% (50) |
|  | Ambohimahasoa(CHL) | Ankafina Tsarafidy | a-cypermethrin |  | ND | 100(100) | S | ND | 25 | ND | 80 | ND | 0% (50) |
|  |  |  | bendiocarb | 100(100) | S | 100(100) | S | 22 | 25 | 81 | 80 | 0% (50) | 0% (50) |
|  |  |  | deltamethrin | 100(100) | S | 100(100) | S | 23 | 26 | 75 | 80 | 0% (50) | 0% (50) |
|  |  |  | l-cyhalothrin | 99(100) | S | 100(100) | S | 23 | 26 | 75 | 80 | 0% (50) | 0% (50) |
|  |  |  | permethrin | 97(100) | P | 68(100) | R | 22 | 26.5 | 80 | 81 | 0% (50) | 0% (50) |
|  |  |  | p-methyl | 100(100) | S | 100(100) | S | 22 | 25 | 80 | 77 | 0% (50) | 0% (50) |
|  | Fianarantsoa II(CHL) | Vohimarina | a-cypermethrin | 0 | ND | 100(100) | S | ND | 23 | ND | 79 | ND | 0% (50) |
|  |  |  | bendiocarb | 100(100) | S | 100(100) | S | 24 | 23 | 81 | 79 | 0% (50) | 0% (50) |
|  |  |  | deltamethrin | 100(100) | S | 100(100) | S | 22 | 24 | 85 | 78 | 0% (50) | 0% (50) |
|  |  |  | l-cyhalothrin | 100(100) | S | 100(100) | S | 23 | 24 | 80 | 78 | 0% (50) | 0% (50) |
|  |  |  | permethrin | 100(100) | S | 100(100) | S | 22.5 | 24 | 85 | 77 | 0% (50) | 0% (50) |
|  |  |  | p-methyl | 100(100) | S | 100(100) | S | 22.5 | 24 | 84 | 77 | 0% (50) | 0% (50) |
|  | Bekily(south sub-desert) | Bekily | a-cypermethrin | 0 | ND | 100(100) | S | ND | 24.5 | ND | 78 | ND | 0% (50) |
|  |  |  | bendiocarb | 100(100) | s | 100(100) | S | 25 | 25 | 79 | 79 | 0% (50) | 0% (50) |
|  |  |  | deltamethrin | 100(100) | S | 98(100) | S | 24.8 | 24.8 | 77 | 77 | 0% (50) | 0% (50) |
|  |  |  | l-cyhalothrin | 85(100) | R | 80(100) | R | 23.8 | 23.8 | 80 | 80 | 0% (50) | 0% (50) |
|  |  |  | permethrin | 80(100) | R | 75(100) | R | 24.5 | 24.5 | 79 | 79 | 0% (50) | 0% (50) |
|  |  |  | p-methyl | 100(100) | S | 100(100) | S | 23.8 | 23.8 | 80 | 80 | 0% (50) | 0% (50) |
|  | Toamasina II ( east coast) | Vohitrambato | a-cypermethrin | 0 | 0 | 97(100) | P | ND | 25.3 | ND | 83 | ND | 0% (50) |
|  |  |  | bendiocarb | 100(100) | S | 100(100) | S | 26 | 26 | 84 | 92 | 0% (50) | 0% (50) |
|  |  |  | deltamethrin | 92(100) | P | 91(100) | P | 26 | 26 | 84 | 83 | 0% (50) | 0% (50) |
|  |  |  | l-cyhalothrin | 99(100) | S | 99(100) | S | 25 | 26 | 92 | 87 | 0% (50) | 0% (50) |
|  |  |  | permethrin | 100(100) | S | 95(100) | P | 25 | 26.2 | 90 | 88 | 0% (50) | 0% (50) |
|  |  |  | p-methyl | 100(100) | S | 100(100) | S | 26.5 | 25.5 | 85 | 87 | 0% (50) | 0% (50) |
|  | Fenerive East( east coast) | Mahambo | a-cypermethrin | 0 | ND | 91(100) | P | ND | 25 | ND | 85 | ND | 0% (50) |
|  |  |  | bendiocarb | 100(100) | S | 100(100) | S | 25 | 25 | 80 | 80 | 0% (50) | 0% (50) |
|  |  |  | deltamethrin | 99(100) | S | 100(100) | S | 26 | 25 | 85 | 80 | 0% (50) | 0% (50) |
|  |  |  | l-cyhalothrin | 99(100) | S | 100(100) | S | 27 | 26 | 85 | 85 | 0% (50) | 0% (50) |
|  |  |  | permethrin | 99(100) | S | 100(100) | S | 27 | 27 | 80 | 85 | 0% (50) | 0% (50) |
|  |  |  | p-methyl | 100(100) | S | 100(100) | S | 26 | 27 | 85 | 80 | 0% (50) | 0% (50) |
|  | Brickaville( east coast) | Ambodifaho | a-cypermethrin | 0 | ND | 100(100) | S | ND | 23.2 | ND | 80 | ND | 0% (50) |
|  |  |  | bendiocarb | 100(100) | S | 100(100) | S | 23 | 23.2 | 86 | 78 | 0% (50) | 0% (50) |
|  |  |  | deltamethrin | 100(100) | S | 100(100) | S | 22 | 23.5 | 83 | 80 | 0% (50) | 0% (50) |
|  |  |  | l-cyhalothrin | 99(100) | S | 100(100) | S | 23 | 24 | 85 | 75 | 0% (50) | 0% (50) |
|  |  |  | permethrin | 100(100) | S | 95(100) | P | 23.5 | 24 | 80 | 77 | 0% (50) | 0% (50) |
|  |  |  | p-methyl | 100(100) | S | 100(100) | S | 23.5 | 23 | 880 | 80 | 0% (50) | 0% (50) |
|  | Vavatenina( east coast) | Vavatenina | a-cypermethrin |  | ND | 99(100) | S | ND | 26.5 | ND | 88 | ND | 0% (50) |
|  |  |  | bendiocarb | 100(100) | S | 100(100) | S | 26 | 26 | 85 | 85 | 0% (50) | 0% (50) |
|  |  |  | deltamethrin | 98(100) | S | 96(100) | P | 26.5 | 26.5 | 84 | 84 | 0% (50) | 0% (50) |
|  |  |  | l-cyhalothrin | 100(100) | S | 99(100) | S | 26.5 | 26.5 | 89 | 86 | 0% (50) | 0% (50) |
|  |  |  | permethrin | 82(100) | R | 89(100) | R | 26.5 | 26.5 | 85 | 89 | 0% (50) | 0% (50) |
|  |  |  | p-methyl | 100(100) | S | 100(100) | S | 26.5 | 26.5 | 83 | 87 | 0% (50) | 0% (50) |
|  | Farafangana (South east) | Manambotra Sud | a-cypermethrin |  | ND | 100(100) | S | ND | 26 | ND | 85 | ND | 0% (50) |
|  |  |  | bendiocarb | 100(100) | S | 100(100) | S | 27 | 27 | 80 | 80 | 0% (50) | 0% (50) |
|  |  |  | deltamethrin | 100(100) | S | 100(100) | S | 26 | 25 | 82 | 85 | 0% (50) | 0% (50) |
|  |  |  | l-cyhalothrin | 100(100) | S | 100(100) | S | 26 | 26 | 88 | 85 | 0% (50) | 0% (50) |
|  |  |  | permethrin | 100(100) | S | 100(100) | S | 27 | 26 | 90 | 85 | 0% (50) | 0% (50) |
|  |  |  | p-methyl | 100(100) | S | 100(100) | S | 27 | 27 | 85 | 80 | 0% (50) | 0% (50) |
|  | Vangaindrano (South east) | Lopary | a-cypermethrin |  | ND | 100(100) | S | ND | 26 | ND | 85 | ND | 0% (50) |
|  |  |  | bendiocarb | 100(100) | S | 100(100) | S | 26 | 26 | 88 | 87 | 0% (50) | 0% (50) |
|  |  |  | deltamethrin | 100(100) | S | 100(100) | S | 25.5 | 25 | 80 | 82 | 0% (50) | 0% (50) |
|  |  |  | l-cyhalothrin | 100(100) | S | 100(100) | S | 27 | 26 | 85 | 87 | 0% (50) | 0% (50) |
|  |  |  | permethrin | 100(100) | S | 100(100) | S | 27 | 27 | 86 | 85 | 0% (50) | 0% (50) |
|  |  |  | p-methyl | 100(100) | S | 100(100) | S | 26 | 26 | 88 | 80 | 0% (50) | 0% (50) |
|  | Note: ND*= Test was not done, RD*= Test results discarded due to doubts on the quality of the papers. | | | | | | | | | | |  |  |
