# Supplementary material for: Insecticide resistance status of three malaria vectors, Anopheles gambiae (s.l.), An. funestus and An. mascarensis, from the south, central and east coasts of Madagascar
Source: Parasit Vectors. 2017 Aug 23;10:396. doi: 10.1186/s13071-017-2336-9 (PMC5569519; doi:10.1186/s13071-017-2336-9)
Supplement: Supplementary file 2 — Mortality rates of An.gambiae (s.l.) field populations exposed to DDT diagnostic dosage between 2013 and 2014 in eight sentinel sites in Madagascar. (PPTX 42 kb) [file 13071_2017_2336_MOESM2_ESM.pptx]

## Slide 1
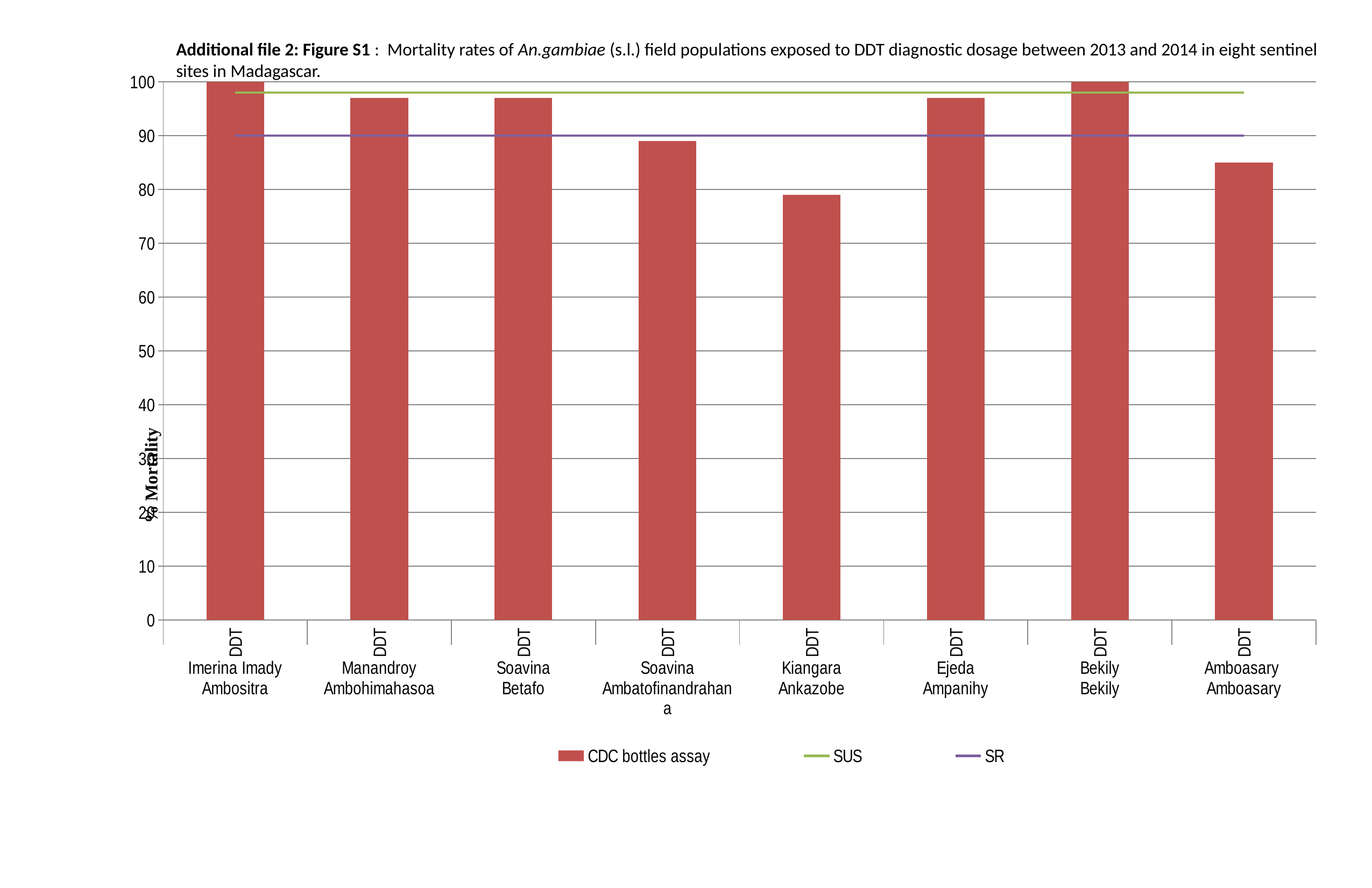

Additional file 2: Figure S1 : Mortality rates of An.gambiae (s.l.) field populations exposed to DDT diagnostic dosage between 2013 and 2014 in eight sentinel sites in Madagascar.
### Chart
| Category | CDC bottles assay | SUS | SR |
|---|---|---|---|
| DDT | 100.0 | 98.0 | 90.0 |
| DDT | 97.0 | 98.0 | 90.0 |
| DDT | 97.0 | 98.0 | 90.0 |
| DDT | 89.0 | 98.0 | 90.0 |
| DDT | 79.0 | 98.0 | 90.0 |
| DDT | 97.0 | 98.0 | 90.0 |
| DDT | 100.0 | 98.0 | 90.0 |
| DDT | 85.0 | 98.0 | 90.0 |
