# Supplementary material for: Insecticide resistance status of three malaria vectors, Anopheles gambiae (s.l.), An. funestus and An. mascarensis, from the south, central and east coasts of Madagascar
Source: Parasit Vectors. 2017 Aug 23;10:396. doi: 10.1186/s13071-017-2336-9 (PMC5569519; doi:10.1186/s13071-017-2336-9)
Supplement: Supplementary file 3 — Mortality rates of An.gambiae (s.l.) field populations exposed to alpha-cypermethrin, permethrin, deltamethrin and lambda-cyhalothrin diagnostic dosages between 2013 and 2014 in nine sentinel sites in Madagascar (PPTX 48 kb) [file 13071_2017_2336_MOESM3_ESM.pptx]

## Slide 1
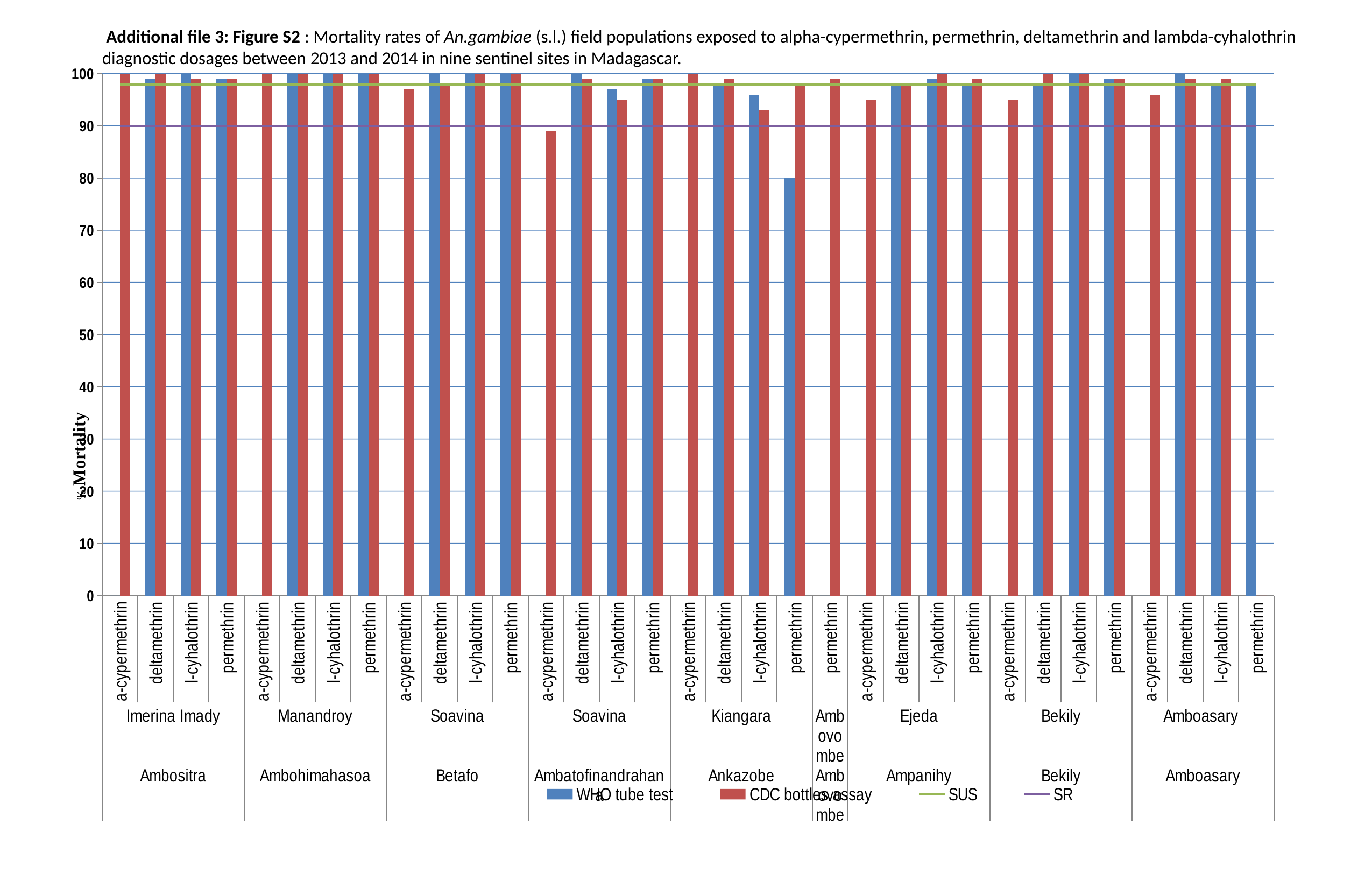

Additional file 3: Figure S2 : Mortality rates of An.gambiae (s.l.) field populations exposed to alpha-cypermethrin, permethrin, deltamethrin and lambda-cyhalothrin diagnostic dosages between 2013 and 2014 in nine sentinel sites in Madagascar.
### Chart
| Category | WHO tube test | CDC bottles assay | SUS | SR |
|---|---|---|---|---|
| a-cypermethrin | 0.0 | 100.0 | 98.0 | 90.0 |
| deltamethrin | 99.0 | 100.0 | 98.0 | 90.0 |
| l-cyhalothrin | 100.0 | 99.0 | 98.0 | 90.0 |
| permethrin | 99.0 | 99.0 | 98.0 | 90.0 |
| a-cypermethrin | 0.0 | 100.0 | 98.0 | 90.0 |
| deltamethrin | 100.0 | 100.0 | 98.0 | 90.0 |
| l-cyhalothrin | 100.0 | 100.0 | 98.0 | 90.0 |
| permethrin | 100.0 | 100.0 | 98.0 | 90.0 |
| a-cypermethrin | 0.0 | 97.0 | 98.0 | 90.0 |
| deltamethrin | 100.0 | 98.0 | 98.0 | 90.0 |
| l-cyhalothrin | 100.0 | 100.0 | 98.0 | 90.0 |
| permethrin | 100.0 | 100.0 | 98.0 | 90.0 |
| a-cypermethrin | 0.0 | 89.0 | 98.0 | 90.0 |
| deltamethrin | 100.0 | 99.0 | 98.0 | 90.0 |
| l-cyhalothrin | 97.0 | 95.0 | 98.0 | 90.0 |
| permethrin | 99.0 | 99.0 | 98.0 | 90.0 |
| a-cypermethrin | 0.0 | 100.0 | 98.0 | 90.0 |
| deltamethrin | 98.0 | 99.0 | 98.0 | 90.0 |
| l-cyhalothrin | 96.0 | 93.0 | 98.0 | 90.0 |
| permethrin | 80.0 | 98.0 | 98.0 | 90.0 |
| permethrin | 0.0 | 99.0 | 98.0 | 90.0 |
| a-cypermethrin | 0.0 | 95.0 | 98.0 | 90.0 |
| deltamethrin | 98.0 | 98.0 | 98.0 | 90.0 |
| l-cyhalothrin | 99.0 | 100.0 | 98.0 | 90.0 |
| permethrin | 98.0 | 99.0 | 98.0 | 90.0 |
| a-cypermethrin | 0.0 | 95.0 | 98.0 | 90.0 |
| deltamethrin | 98.0 | 100.0 | 98.0 | 90.0 |
| l-cyhalothrin | 100.0 | 100.0 | 98.0 | 90.0 |
| permethrin | 99.0 | 99.0 | 98.0 | 90.0 |
| a-cypermethrin | 0.0 | 96.0 | 98.0 | 90.0 |
| deltamethrin | 100.0 | 99.0 | 98.0 | 90.0 |
| l-cyhalothrin | 98.0 | 99.0 | 98.0 | 90.0 |
| permethrin | 98.0 | 0.0 | 98.0 | 90.0 |
