# Supplementary material for: Insecticide resistance status of three malaria vectors, Anopheles gambiae (s.l.), An. funestus and An. mascarensis, from the south, central and east coasts of Madagascar
Source: Parasit Vectors. 2017 Aug 23;10:396. doi: 10.1186/s13071-017-2336-9 (PMC5569519; doi:10.1186/s13071-017-2336-9)
Supplement: Supplementary file 5 — Anopheles gambiae (s.l.) insecticide susceptibility test results with and without pre-exposure to synergists by site/village using CDC bottle bioassays (DOCX 18 kb) [file 13071_2017_2336_MOESM5_ESM.docx]

**Additional file 5: Table S2.** *Anopheles gambiae* (*s.l.*) insecticide susceptibility test results with and without pre-exposure to synergists by site/village using CDC bottle bioassys.

| Site (eco-epidemiological zone) | Insecticide tested | # tested | # dead | % mortality | Resistance status |
| --- | --- | --- | --- | --- | --- |
| Imerina Imady (CHL) | L-cyhalothrin | 150 | 142 | 95% | PR |
|  | L-cyhalothrin + PBO | 150 | 150 | 100% | S |
|  | PBO | 50 | 0 | 0% |  |
|  | Control | 75 | 0 | 0% |  |
| Ankafina-Tsarafidy(CHL) | Permethrin | 150 | 107 | 71% | R |
|  | Permethrin +PBO | 150 | 150 | 100% | S |
|  | Permethrin + DEF | 100 | 91 | 91% | PR |
|  | PBO | 50 | 0 | 0% |  |
|  | Control | 75 | 0 | 0% |  |
| Vavatenina (EC) | Permethrin | 150 | 133 | 89% | R |
|  | Permethrin +PBO | 150 | 150 | 100% | S |
|  | Permethrin + DEF | 100 | 92 | 92% | PR |
|  | PBO | 50 | 0 | 0% |  |
|  | Control | 75 | 0 | 0% |  |
|  |  |  |  |  |  |
|  | Deltamethrin | 150 | 141 | 94% | PR |
|  | Deltamethrin +PBO | 150 | 150 | 100% | S |
|  | Deltamethrin + DEF | 100 | 100 | 100% | S |
|  | PBO | 50 | 0 | 0% |  |
|  | Control | 75 | 0 | 0% |  |
| Bekily (SE) | Permethrin | 100 | 75 | 75% | R |
|  | Permethrin +PBO | 100 | 100 | 100% | S |
|  | Control | 50 | 0 | 0% |  |
| Ambodifaho( EC) | Permethrin | 150 | 141 | 94% | PR |
|  | Permethrin +PBO | 150 | 146 | 97% | PR |
|  | PBO | 50 | 0 | 0% |  |
|  | Control | 75 | 0 | 0% |  |
| Vohitrambato (EC) | Permethrin | 150 | 140 | 93% | PR |
|  | Permethrin +PBO | 150 | 150 | 100% | S |
|  | PBO | 50 | 0 | 0% |  |
|  | Control | 75 | 0 | 0% |  |
|  |  |  |  |  |  |
|  | Deltamethrin | 150 | 138 | 92% | PR |
|  | Deltamethrin +PBO | 150 | 150 | 100% | S |
|  | Deltamethrin + DEF | 100 | 100 | 100% | S |
|  | PBO | 50 | 0 | 0% |  |
|  | Control | 75 | 0 | 0% |  |
|  |  |  |  |  |  |
|  | a-cypermethrin | 100 | 91 | 91% | PR |
|  | a-cypermethrin +PBO | 100 | 100 | 100% | S |
|  | Control | 75 | 0 | 0% |  |
| Mahambo (EC) | Permethrin | 150 | 125 | 83% | R |
|  | Permethrin +PBO | 150 | 150 | 100% | S |
|  | Permethrin + DEF | 100 | 100 | 100% | S |
|  | PBO | 50 | 0 | 0% |  |
|  | Control | 75 | 0 | 0% |  |
|  |  |  |  |  |  |
|  | a-cypermethrin | 150 | 136 | 91% | PR |
|  | a-cypermethrin +PBO | 150 | 150 | 100% | S |
|  | a-cypermethrin + DEF | 100 | 100 | 100% | S |
|  | PBO | 50 | 0 | 0% |  |
|  | Control | 75 | 0 | 0% |  |
